# Combining Genes from Multiple Phages for Improved Cell Lysis and DNA Transfer from *Escherichia coli* to *Bacillus subtilis*

**DOI:** 10.1371/journal.pone.0165778

**Published:** 2016-10-31

**Authors:** Mario Juhas, Christine Wong, James W. Ajioka

**Affiliations:** Department of Pathology, University of Cambridge, Cambridge, United Kingdom; Loyola University Chicago, UNITED STATES

## Abstract

The ability to efficiently and reliably transfer genetic circuits between the key synthetic biology chassis, such as *Escherichia coli* and *Bacillus subtilis*, constitutes one of the major hurdles of the rational genome engineering. Using lambda Red recombineering we integrated the thermosensitive lambda repressor and the lysis genes of several bacteriophages into the *E*. *coli* chromosome. The lysis of the engineered autolytic cells is inducible by a simple temperature shift. We improved the lysis efficiency by introducing different combinations of lysis genes from bacteriophages lambda, ΦX174 and MS2 under the control of the thermosensitive lambda repressor into the *E*. *coli* chromosome. We tested the engineered autolytic cells by transferring plasmid and bacterial artificial chromosome (BAC)-borne genetic circuits from *E*. *coli* to *B*. *subtilis*. Our engineered system combines benefits of the two main synthetic biology chassis, *E*. *coli* and *B*. *subtilis*, and allows reliable and efficient transfer of DNA edited in *E*. *coli* into *B*. *subtilis*.

## Introduction

The ability to efficiently and reliably transfer genetic circuits between different synthetic biology chassis, such as *Escherichia coli* and *Bacillus subtilis*, constitutes one of the main bottlenecks of the rational genome engineering. The Gram-negative *E*. *coli* and the Gram-positive *B*. *subtilis* are both well-characterized bacteria used in a number of synthetic biology and biotechnology applications [1–4]. Furthermore, they are considered to be promising chassis for the construction of the minimal cell factories [1, 5–7]. *E*. *coli* is easily amenable to genetic modifications by a number of DNA recombineering methods. Although *E*. *coli* was successfully engineered for the production of a number of industrially relevant products, such as biofuels, amino acids and isoprenoids [2, 8–10], *B*. *subtilis* is considered to be a better host for certain applications [11–13]. Unlike *E*. *coli*, *B*. *subtilis* is naturally competent and readily transformable with extracellular DNA which is integrated into the chromosome via RecA-mediated homologous recombination [14, 15]. Furthermore, *B*. *subtilis* secretes proteins into the medium and forms durable endospores.

Novel tools combining benefits of the *B*. *subtilis* chassis with the reliable DNA recombineering in *E*. *coli* are therefore crucial for *B*. *subtilis* bioengineering efforts. Due to natural competence of *B*. *subtilis*, DNA edited in and released from the lysed *E*. *coli* cells can be readily taken up and incorporated into the *B*. *subtilis* chromosome. On the industrial scale, the conditionally-inducible cell lysis systems are preferable over the traditional methods of cell lysis, such as enzyme degradation or mechanical disruption for a number of reasons, such as lower production and product recovery costs and no need for an inducer [16]. Transfer of DNA from *E*. *coli* undergoing lambda prophage-induced lysis to *B*. *subtilis* by the co-culture method was demonstrated recently [17, 18]. The co-culture method was also shown to be suitable for transferring high molecular weight DNA molecules, which are difficult to manipulate with other methods due to mechanical shearing [17, 18]. However, the presence of an entire phage in the donor cell can be detrimental to the quality of the transferred DNA. This problem can be minimized by using solely the phage lysis genes.

Here we present a system for the reliable and efficient DNA transfer that utilizes the lysis genes from multiple phages to disrupt the donor *E*. *coli* cell and transfer the released DNA into *B*. *subtilis*.

## Materials and Methods

### Bacterial strains, BACs, plasmids, and growth conditions

All bacterial strains, plasmids and BACs used in this study are listed in Table 1. *Escherichia coli* and *Bacillus subtilis* were routinely grown in Luria-Bertani broth (LB). When required, cultivation media were supplemented with chloramphenicol (30 μg/ml), ampicillin (100 μg/ml) and kanamycin (50 μg/ml) for growing *E*. *coli*, and chloramphenicol (5 μg/ml) and kanamycin (5 μg/ml) for *B*. *subtilis*. Liquid cultures were grown in LB on a rotatory shaker at 200 r.p.m. and 30°C, 37°C or 42°C, as required. Plate cultures were incubated at 30°C, 37°C or 42°C for approximately 24 hours. Starvation medium (described below) was used to generate competent *B*. *subtilis* cells.

**Table 1:** Bacterial strains, BACs and plasmids used in this study.

	Characteristics	Reference
**Strains**		
Ec	*E*. *coli* wild type strain K12 MG1655	[19]
Bs	*B*. *subtilis* wild type strain 168	Lab collection
Ec(Ri)	*E*. *coli* with ts repressor integrated	[20]
Ec(RL1i)	*E*. *coli* with ts repressor and *MS2* integrated	This study
Ec(RL2i)	*E*. *coli* with ts repressor and *ΦX174* integrated	This study
Ec(RL3i)	*E*. *coli* with ts repressor and *λ* integrated	This study
Ec(RL12i)	*E*. *coli* with ts repressor and *MS2*,*ΦX174* integr	This study
Ec(RL13i)	*E*. *coli* with ts repressor and *MS2*, *λ* integr	This study
Ec(RL123i)	*E*. *coli* with ts repressor and *MS2*,*ΦX174*, *λ* integr	This study
Ec(RL1)	*E*. *coli* with ts repressor and pSB1K3(FRTL1)	This study
Ec(RL2)	*E*. *coli* with ts repressor and pSB1K3(FRTL2)	This study
Ec(RL3)	*E*. *coli* with ts repressor and pSB1K3(FRTL3)	This study
Ec(RL12)	*E*. *coli* with ts repressor and pSB1K3(FRTL12)	This study
Ec(RL13)	*E*. *coli* with ts repressor and pSB1K3(FRTL13)	This study
Ec(RL123)	*E*. *coli* with ts repressor and pSB1K3(FRTL123)	This study
Bs(cav)	*B*. *subtilis* with integrated *cat/ven* circuits	This study
**Plasmids and BACs**		
pSB1K3	standard BioBrick assembly plasmid	Parts registry
pKM208	plasmid with IPTG-inducible λ red system	[21]
pCP20	plasmid with FLP recombinase	[22]
pSB1K3(FRTKr)	ts repressor in pSB1K3	[20]
pJScav	*cat*,*ven* circuits	Parts registry
pSB1K3(FRTL1)	*MS2* lysis gene in pSB1K3	This study
pSB1K3(FRTL2)	*ΦX14* lysis gene in pSB1K3	This study
pSB1K3(FRTL3)	*λ* lysis gene in pSB1K3	This study
pSB1K3(FRTL12)	*MS2* and *ΦX14* lysis genes in pSB1K3	This study
pSB1K3(FRTL13)	*MS2* and *λ* lysis genes in pSB1K3	This study
pSB1K3(FRTL123)	*MS2*, *ΦX14* and *λ* lysis genes in pSB1K3	This study
iBAC(cav)g	pBeloBAC11, *amyE* sites, *ven/cat* circuits	This study

### Competent *B*. *subtilis* generation and transformation

To generate competent *B*. *subtilis* cells, single colony was first inoculated into 10 ml minimal medium composed of 5x minimal salts solution [ammonium sulphate (10 mg/ml), potassium hydrogen phosphate (75 mg/ml), potassium dihydrogen phosphate (25 mg/ml), sodium citrate (1 mg/ml), magnesium sulphate heptahydrate (1mg/ml)], glucose (0.5% w/v), casamino acids (0.02% w/v), tryptophan (20 μg/ml), and iron ammonium citrate (2.2 μg/ml). Inoculated cells were grown at 200 r.p.m. on a rotatory shaker at 37°C for 18 hours. Then, 1.4 ml of the *B*. *subtilis* culture was inoculated into 10 ml of the fresh minimal medium and grown for another 3 hours. Subsequently, 11 ml of the starvation medium composed of 5x minimal salts solution and glucose (0.5% w/v) were added to the *B*. *subtilis* culture and cells were grown for additional 2 hours and 45 mins. 0.3 ml aliquots were transferred into 15 ml polypropylene tubes and transformed with 15 μl of BAC DNA or plasmid DNA. Transformed *B*. *subtilis* cells were incubated at 200 r.p.m. on a rotatory shaker at 37°C for 1 hour prior to addition of 700 μl LB. Cells were then continued to grow for 1.5–2 hours. 20–200 μl of this culture were plated onto selection plates and grown at 37°C for 18–24 hours. Integrations into the *B*. *subtilis* chromosome were confirmed by PCR with the flanking primers and sequencing.

### PCR amplification and DNA modification methodology

PCR amplifications were performed in 25 or 50 μl reaction mixtures using Phusion DNA polymerase (Thermo Scientific) or Dream Taq master mix kit (Thermo Scientific) according to the supplier’s instructions. Oligonucleotide primers were synthesized by Sigma-Aldrich. PCR amplified DNA fragments were assembled in a 5.2 μl final reaction volume using the modified Gibson Assembly procedure [20, 23, 24]. DNA assemblies were confirmed by PCR amplification and sequencing. DNA sequencing was performed by Source Bioscience. Qiaquick Gel Extraction kit (Qiagen) and Qiaprep Spin Miniprep kit (Qiagen) were used for DNA extraction and purification and plasmid isolation, respectively. BACs were isolated with Qiaquick Gel Extraction kit (Qiagen) or PhasePrep BAC DNA kit (Sigma-Aldrich), according to the manufacturer’s instructions. *B*. *subtilis* genomic DNA was obtained using the GeneJET genomic DNA purification kit (Thermo Scientific).

### Lambda Red recombineering for *E*. *coli* chromosomal integration

The modified Miller and Nickoloff [25] and Hanahan [26] methods were used to generate the electro-competent and chemically competent *E*. *coli*, respectively. DNA integrations into *E*. *coli* chromosome and BACs were performed using the streamlined lambda Red system-mediated method described previously [20, 27, 28]. Briefly, *E*. *coli* was transformed with pKM208-bourne IPTG-inducible lambda red recombinase and grown on the selective ampicillin plates at 30°C overnight. Overnight culture (1:100 dilution) was inoculated into LB with ampicillin and grown at 30°C first to OD_600_ 0.2. Then, IPTG (1 mM) was added and the culture was grown to the final OD_600_ 0.5. Cells were washed and resuspended in 100 μl of 10% glycerol per 100 ml of starting culture volume. 100 μl of the PCR amplified and gel purified DNA flanked by sequences homologous to the integration target loci were electroporated into *E*. *coli* with pKM208. Transformants were plated on selective plates and grown at 30°C overnight and then grown on LB plates at 42°C. As pKM208 is thermosensitive, growth at 42°C leads to loss of this plasmid from *E*. *coli* cells. DNA integrations were confirmed by diagnostic PCR with flanking primers and DNA sequencing. To allow repeated use of the kanamycin resistance marker, cells with DNA integrations were transformed with plasmid pCP20 encoding flippase (FLP) recombinase and grown first on selective plates at 30°C overnight and then grown on LB plates at 42°C to cure out the thermosensitive pCP20.

### Culture mix method of DNA transfer from *E*. *coli* to *B*. *subtilis*

Modified culture mix method [17, 18] was used for DNA transfer from *E*. *coli* to *B*. *subtilis*. Briefly, donor *E*. *coli* was grown in LB at 30°C for 24 hours and then diluted (1:200) in a pre-warmed 20 ml LB supplemented with antibiotics and grown at 30°C for 5 hours. *E*. *coli* culture was harvested by centrifugation at 5000 r.p.m. for 8 mins, re-suspended in a fresh pre-warmed 20 ml LB and grown for another 1 hour. The recipient *B*. *subtilis* was grown in LB at 37°C for 17 hours, diluted (1:200) in a pre-warmed 20 ml TFI medium [5 x minimal salts solution, glucose (0.5% w/v), tryptophan (50 μg/ml), arginine (50 μg/ml), leucine (50 μg/ml), threonine (50 μg/ml), casamino acids (2% w/v)] and grown at 37°C for 5 hours. The donor *E*. *coli* and the recipient *B*. *subtilis* cultures were mixed (mixing ratio 1:1) and cultivated at 42°C for 2 and 6 hours. 200 μl of the mixture was spread on LB plate with chloramphenicol to select for *B*. *subtilis* transformants. Residual *E*. *coli* was removed by inducing *B*. *subtilis* sporulation and subsequent heat treatment to kill the growing cells. Briefly, colonies scraped off the plates were resuspended in 1 ml 2 x SG medium [Difco nutrient broth (Difco Laboratories) (16 mg/ml), KCl (2 mg/ml), MgSO_4_.7H_2_O (0.5 mg/ml), 1M Ca(NO_3_)_2_ (0.1% v/v), 0.1M MnCl_2_.4H_2_O (0.1% v/v), 1mM FeSO_4_ (0.1% v/v), glucose (0.1% w/v)] and grown on 2 x SG plates for 72 hours to induce sporulation. Bacteria scraped off the 2 x SG plates were washed with ice-cold water and subjected to heat treatment at 90°C for 10 minutes. 100 μl were plated on LB chloramphenicol plates and grown overnight. The average numbers of CFUs and standard errors were calculated from three independent replicates.

### Bacterial viability assay

The *E*. *coli* viability was measured with a Live/Dead BacLight^™^ bacterial viability kit (Lifetechnologies, UK) according to supplier’s instructions. Briefly, cells were first grown in 5 ml LB in 15 ml Falcon conical centrifuge tubes on a rotatory shaker at 200 r.p.m. and 30°C for 6 hours prior to temperature shift to 42°C. After the temperature shift cells were grown at 42°C for another 6 hours. Then red-fluorescent nucleic acid stain propidium iodide and green-fluorescent nucleic acid stain SYTO9 were added into the medium (3 μl of the stain per ml). The numbers of the dead propidium iodide-stained bacteria and live SYTO-9 stained bacteria were determined by fluorescent microscope (Nikon Microphot-SA), 5–6 hours after the temperature shift from 30°C to 42°C. The means and standard errors were calculated from three experiments.

### Absorbance measurement with the microplate reader

Absorbance was measured in the 96 well microplates (clear, flat-bottomed, Sterilin Sero-Well, UK). Bacterial cultures were grown overnight and normalized to OD_600_ of 0.05. 200 μl were aliquoted into the microplate wells and incubated in the microplate reader (Fluostar Omega, BMG Labtech, UK) at 30°C for 24 hours or at 30°C for 3 hours, followed by incubation at 42°C for another 21 hours. Absorbance was measured with the automatically repeated protocol (absorbance filter 600 nm, cycle time 60 min, double orbital shaking at 500 r.p.m.).

### Databases and sequence analyses

BLASTN [29] and TBLASTX algorithms and position-specific iterated BLAST (PSI-BLAST) [30] of the National Centre for Biotechnology Information (NCBI) website (http://ncbi.nlm.nih.gov) were used to compare DNA sequences. *E*. *coli* K-12 project website (http://www.xbase.ac.uk/genome/escherichia-coli-str-k-12-substr-mg1655) was the source of the *E*. *coli* genome sequence. BioCyc (http://bsubcyc.org/) and SubtiWiki (http://subtiwiki.uni-goettingen.de/) databases were used to obtain the *B*. *subtilis* genome sequence. The sequences of DNA constructs were obtained from the Registry of Standard Biological Parts (http://parts.igem.org/Main_Page), the NCBI website and the Addgene non-profit plasmid repository (http://www.addgene.org/). Sequencing was done by Source Bioscience.

## Results and Discussion

### Lysis genes of phages MS2, ΦX174, and lambda for cell lysis

Bacteriophages MS2, ΦX174 and lambda were chosen mainly due to their extensive characterization and different mechanisms of action [31]. In *E*. *coli*, there are 3 main barriers to phage release, namely an outer membrane (OM), a thin layer of peptidoglycan (PG), and a cell membrane (CM). The lysis gene cassette of the phage lambda consists of four genes, namely *S*, *R*, *Rz* and *Rz1* regulated by *pR* promoter. *Rz* and *Rz1* encode a class II CM protein and an OM lipoprotein, respectively, which form a complex spanning the periplasm of the host cell to promote fusion of the CM with the OM [32]. *S* encodes the holin and the antiholin [33], while *R* encodes the endolysin with lytic transglycosylase activity [34]. The endolysin and the holin accumulate in the cytoplasm and the CM, respectively. The holin-induced disruption of the CM allows endolysin-driven deterioration of the PG and cell lysis [35]. The lysis gene of the phage ΦX174 encodes E protein implicated in the inhibition of the PG biosynthesis. This leads to the cell wall rupture at the developing septum. As a consequence, the cell lysis induced by phage ΦX174 is more gradual than that of the phage lambda [36]. The lysis gene of the phage MS2 encodes L protein that binds to the adhesion zones formed by parts of the CM, the OM and the periplasm. As formation of the adhesion zones is dependent on the activation of the cell‘s autolysis system, the MS2-induced lysis occurs in the actively growing cells [37–39]. The different mechanisms of action of the lambda, ΦX174 and MS2 lysis genes suggests that their combination in a single cell could lead to more efficient lysis.

### Engineering the inducible lysis system

**Fig 1:**
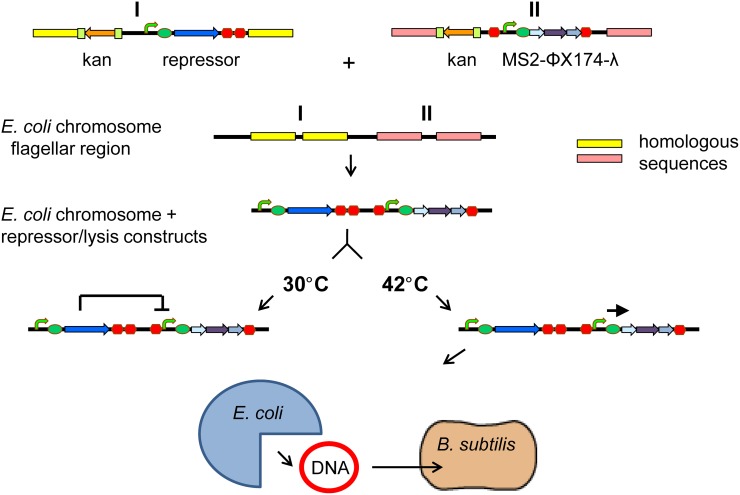
The inducible cell lysis system. Figure depicts schematic view of the engineered cell lysis system. The thermosensitive lambda repressor and lysis genes from bacteriophages MS2, ΦX174, and lambda whose expression is controlled by *pL* promoter were integrated into the flagellar region of the *E*. *coli* K12 MG1655 chromosome. The kanamycin resistance marker for selection of cells with the chromosomally integrated DNA is flanked by flippase recognition target (FRT) sites. The thermosensitive lambda repressor was integrated into the chromosome first. After integration of the lambda repressor, the kanamycin resistance marker was "flipped out" from the chromosome using flippase (FLP) recombinase to allow repeated use of the kanamycin for selection of the second construct encoding lysis genes. At restrictive temperature (30°C), repressor inhibits expression of phage lysis genes. At permissive temperature (42°C), expression of lysis genes leads to the cell lysis and release of DNA from *E*. *coli*. Released DNA can be then taken up by other bacteria, such as *B*. *subtilis*. Kan: kanamycin; repressor: thermosensitive lambda repressor; MS2: lysis gene of the phage MS2; ΦX174: lysis gene of the phage ΦX174; λ: lysis gene of the phage lambda.

To construct an inducible system for controlled *E*. *coli* lysis we chose the thermosensitive lambda repressor to regulate the expression of the phage lambda, ΦX174 and MS2 lysis genes. At 30°C, the expression of lysis genes from the *pL* promoter is inhibited by the repressor. Temperature shift to 42°C alleviates the repression and leads to cell lysis. DNA can be then taken up by other bacteria, such as *B*. *subtilis* (Fig 1).

To construct the inducible cell lysis system we first engineered genetic circuits Repr-ts-1, L1, L2, L3, L12, L13, and L123. The genetic circuit Repr-ts-1 consists of the thermosensitive lambda repressor, strong constitutive promoter, RBS and terminator from the Registry of Standard Biological Parts (http://parts.igem.org/Main_Page) [20] (Fig 1). The genetic circuits L1, L2, L3, L12, L13, and L123 encode lysis genes of the bacteriophages MS2, ΦX174, lambda, MS2+ΦX174, MS2+lambda, and MS2+ΦX174+lambda, respectively. Lysis genes in genetic circuits L1, L2, L3, L12, L13, and L123 are located downstream of the *pL* promoter regulated by the lambda repressor (Fig 1). In addition to lysis genes and *pL* promoter, the genetic circuits L1, L2, L3, L12, L13, and L123 harbour a RBS in front of the first coding sequence and are isolated on both ends with the T0 and t1 transcriptional terminators from the Registry of Standard Biological Parts (Fig 1). We constructed strain Ec(Ri) by integrating the genetic circuit Repr-ts-1 into the *E*. *coli* chromosome using streamlined lambda Red recombinase method [20, 22, 27, 28]. The kanamycin resistance marker was "flipped out" from Ec(Ri) using flippase (FLP) recombinase. To test the inducible cell lysis system, we first introduced the genetic circuits L1, L2, L3, L12, L13, and L123 into strain Ec(Ri) on plasmids pSB1K3(FRTL1), pSB1K3(FRTL2), pSB1K3(FRTL3), pSB1K3(FRTL12), pSB1K3(FRTL13), and pSB1K3(FRTL123), respectively. Integration of genetic circuits into the chromosome is preferable to their maintenance on the plasmids [40, 41], therefore, in the next step we also integrated L1, L2, L3, L12, L13, and L123 into the chromosome of the strain Ec(Ri). The correct assemblies of lysis genes on plasmids and the chromosomally integrated genetic circuits were verified by diagnostic PCR with flanking primers (S1 Fig and S1 Table) and sequencing.

### Combining lysis genes from multiple phages improves cell lysis

To test the effect of multiple phage lysis genes on cell lysis we first used plasmids pSB1K3(FRTL1), pSB1K3(FRTL2), pSB1K3(FRTL3), pSB1K3(FRTL12), pSB1K3(FRTL13), and pSB1K3(FRTL123), harbouring different combinations of lysis genes (Table 1). The cell lysis was quantified by measuring absorbance of the *E*. *coli* cultures with the microplate reader. The absorbance of the bacterial cultures grown in both restrictive and permissive temperature for the thermosensitive repressor was compared with that of the controls grown in the restrictive temperature for 24 hours. The absorbance was significantly lower after the temperature shift when compared to the growth in the restrictive conditions for the whole 24 hours. The comparison between cultures harbouring plasmids with different combinations of lysis genes showed that the strain with lysis genes from all three bacteriophages lysed with the highest efficiency. Furthermore, lysis of strains harbouring two lysis genes was higher than that harbouring a single lysis gene (data not shown). The highest difference between restrictive and restrictive/permissive conditions was observed for *E*. *coli* with plasmid pSB1K3(FRTL123) encoding all three lysis genes.

**Fig 2:**
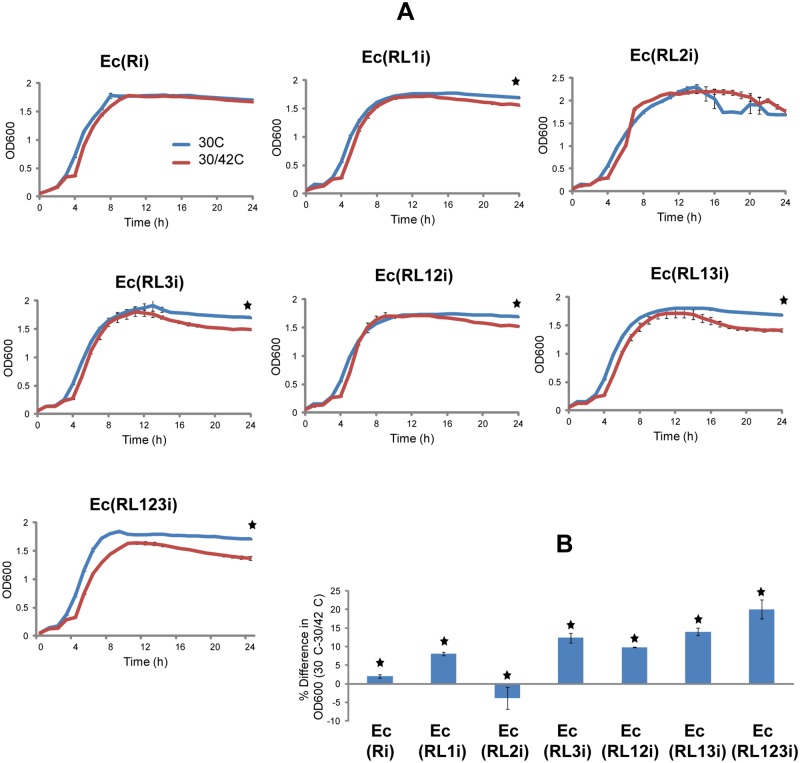
Lysis efficiency measured with the microplate reader. **(A)** Figure shows cell lysis efficiencies measured as absorbance over time with the microplate reader for strain Ec(Ri) and strains Ec(RL1i), Ec(RL2i), Ec(RL3i), Ec(RL12i), Ec(RL13i), and Ec(RL123i) harbouring integrated genetic circuits L1, L2, L3, L12, L13 and L123, respectively. Cells were grown at the restrictive conditions for the repressor for 24 hours (30°C) and at the restrictive/permissive conditions (restrictive conditions for 3 hours, followed by the permissive conditions for additional 21 hours (30/42°C)). Experiments were carried out in triplicate and the mean and standard error were calculated. T-tests were conducted to compare the differences between the absorbance at the restrictive (30°C) and restrictive/permissive conditions (30/42°C) at the end of measurement period for each analyzed strain. Stars show significant difference (p<0.05) between the absorbance at 30°C and 30/42°C for each strain. The differences were significant in all strains, except Ec(Ri) and Ec(RL2i). **(B)** Figure shows combinatorial effect of lysis genes on the degree of lysis calculated from the differences between the mean absorbances of cultures grown at 30°C and 30/42°C at the end of measurement period. T-tests were performed to compare the differences between strains Ec(RL123i) and other strains harbouring fewer lysis genes. Stars indicate significant differences (p<0.05) in the lysis efficiencies between Ec(RL123i) and other strains.

Next, to verify that the observed differences were not due to the variable plasmid copy numbers, genetic circuits L1, L2, L3, L12, L13 and L123 were integrated into the chromosome of the *E*. *coli* strain Ec(Ri). The lysis efficiencies of the engineered strains Ec(RL1i), Ec(RL2i), Ec(RL3i), Ec(RL12i), Ec(RL13i), and Ec(RL123i) with integrated lysis genes were first calculated from the difference between the absorbance at the restrictive and restrictive/permissive conditions measured with the microplate reader (Fig 2A and 2B). The statistical T-test analysis showed that there were significant differences between the absorbance at restrictive and restrictive/permissive conditions in strains Ec(RL1i) (p<0.005), Ec(RL3i) (p<0.005), Ec(RL12i) (p<0.005), Ec(RL13i) (p<0.005) and Ec(RL123i) (p<0.005) (Fig 2A). The differences were not statistically significant (p>0.05) in strains Ec(Ri) and Ec(RL2i) (Fig 2B). To assess the combinatorial effect of lysis genes on the degree of lysis we calculated the differences between the mean absorbances of the strain Ec(RL123i) and other strains harbouring fewer lysis genes grown at restrictive and restrictive/permissive conditions (Fig 2B). The statistical T-test analysis showed that there were significant differences between the lysis effect of Ec(RL123i) and Ec(RL1i), Ec(RL123i) and Ec(RL2i), Ec(RL123i) and Ec(RL3i), Ec(RL123i) and Ec(RL12i), and Ec(RL123i) and Ec(RL13i) with 12% (p<0.005), 20% (p<0.005), 8% (p<0.05), 10% (p<0.01) and 6% (p<0.05) increase in the absorbance difference, respectively (Fig 2B). The lysis efficiency increased with the number of the chromosomally integrated phage lysis genes (Fig 2B), thus confirming results obtained with plasmids pSB1K3(FRTL1), pSB1K3(FRTL2), pSB1K3(FRTL3), pSB1K3(FRTL12), pSB1K3(FRTL13), and pSB1K3(FRTL123). The highest lysis efficiency was observed in strain Ec(RL123i) harbouring all three lysis genes (Fig 2B).

In our experimental setting, the drop in the absorbance in strains Ec(RL1i), Ec(RL2i), Ec(RL3i), Ec(RL12i) and Ec(RL13i) occurred after approximately 9 hours after induction of the phage genes. Only in the strain Ec(RL123i) harbouring all three lysis genes the effect of the cell lysis on the absorbance was clearly visible after approximately 4 hours after induction. The differences in the kinetics of the cell lysis can be attributed to the variable gene components used in cell lytic systems. The previously developed systems utilized entire phages to lyse the *E*. *coli* cell [17, 18]. The entire lambda phage genome has approximatelly 48 kb and in addition to lysis genes encodes a number of proteins crucial for the phage's life cycle. Furthermore, following induction, genome of the wild type lambda phage starts replicating and around 100 virions are released from each cell after lysis [42]. In comparison, our cell lytic system relies solely on individual phage lysis genes which are stably integrated into the host cell's chromosome. Thus there is only a single copy of individual lysis genes in each cell and there are no other phage-encoded proteins present that contribute to the phage's life cycle.

**Fig 3:**
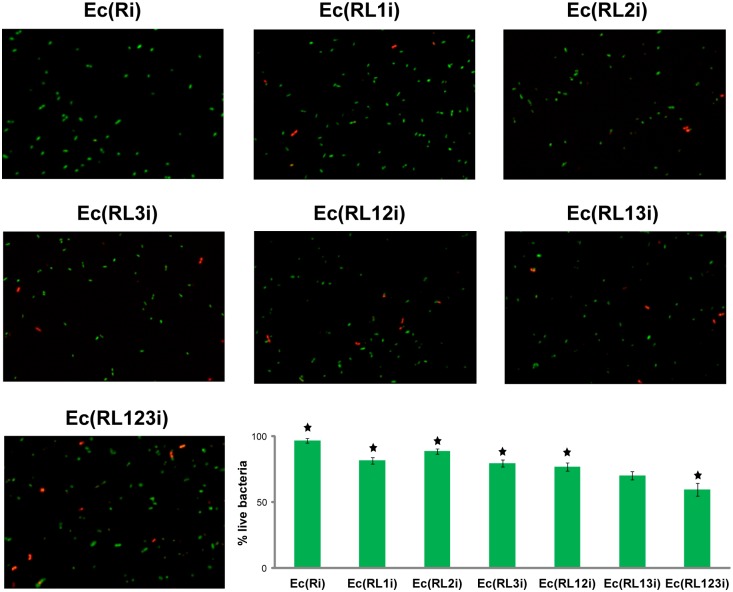
Fluorescent microscopy and live/dead staining. Live/dead staining of strain Ec(Ri) and strains Ec(RL1i), Ec(RL2i), Ec(RL3i), Ec(RL12i), Ec(RL13i), and Ec(RL123i) harbouring integrated genetic circuits L1, L2, L3, L12, L13 and L123, respectively. Green fluorescence shows viable bacteria with intact cell membranes, while red fluorescence indicates dead cells with damaged membranes. The numbers of the dead and live bacteria were determined 5–6 hours after the temperature shift from 30°C to 42°C. The survival of Ec(RL123i) was most reduced when compared to the wild type Ec(Ri). The means and standard errors were calculated from three experiments. T-tests were performed to compare the differences in the proportion of live bacteria between Ec(RL123i) and strains harbouring fewer lysis genes. Stars indicate significant difference in the percentage of live bacteria between Ec(RL123i) and the analyzed strain. The differences in the proportion of live bacteria were statistically significant (p<0.05) in all strains with the exception of Ec(RL13i).

The outcome of the absorbance measurement can be influenced by a number of factors (e.g. debris from the lysed cells). Therefore, to confirm the results of absorbance assays, lysis efficiencies were also investigated on the level of the individual cells. Bacteria grown at the restrictive and permissive conditions were stained with the *Bac*Light Live/Dead bacterial viability stain and examined by fluorescent microscopy. The survival rates of strains Ec(RL1i), Ec(RL2i), Ec(RL3i), Ec(RL12i), Ec(RL13i), and Ec(RL123i) after temperature shift were significantly reduced when compared to the *E*. *coli* strain with no lysis genes Ec(Ri) (Fig 3). Furthermore, the statistical analysis showed that there were significant differences in the proportion of live bacteria between Ec(RL123i) and strains harbouring fewer lysis genes [Ec(RL1i) (p<0.01), Ec(RL2i) (p<0.005), Ec(RL3i) (p<0.05), Ec(RL12i) (p<0.05)], with the exception of Ec(RL13i) (p>0.05) (Fig 3). The survival of Ec(RL123i) was most affected (Fig 3), thus confirming that combining lysis genes from bacteriophages MS2, ΦX174, and lambda in a single *E*. *coli* cell improves lysis.

### DNA transfer from *E*. *coli* to *B*. *subtilis* by induced cell lysis

**Fig 4:**
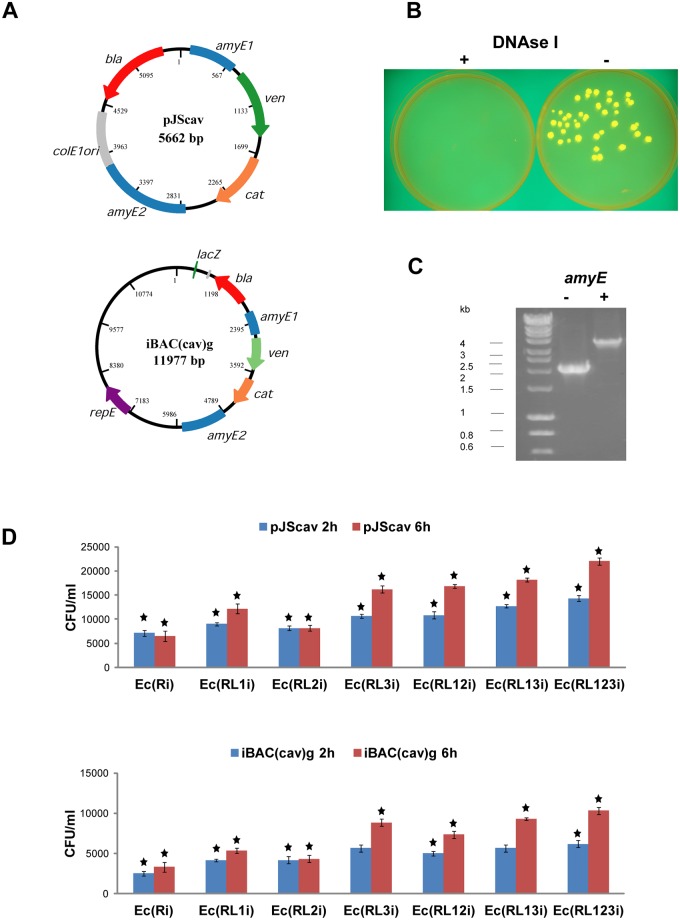
Interchassis DNA transfer from *E*. *coli* to *B*. *subtilis* by induced cell lysis. **(A)** pJSav and iBAC(cav) maps. pJSav and iBAC(cav) encode genetic circuits *cat* and *ven* flanked by the integration sequences homologous to *amyE* target site in the *B*. *subtilis* chromosome. *cat* and *ven* encode *Bacillus*-specific chloramphenicol resistance and yellow fluorescent protein gene *mVenus*, respectively. **(B)** DNAse I suppressed DNA transfer from *E*. *coli* to *B*. *subtilis* in the culture media. **(C)** Integration of the pJSav and iBAC(cav)-borne genetic circuits *cat* and *ven* into *amyE* locus of the *B*. *subtilis* chromosome. HyperLadder 1kb (Bioline) has been used as the molecular weight marker. **(D)** Number of *B*. *subtilis* transformants per ml of mixed *E*. *coli* and *B*. *subtilis* cultures using pJSav and iBAC(cav) 2 hours and 6 hours after temperature induced lysis. The number of transformants was highest with the strain Ec(RL123i) harbouring lysis genes from all three bacteriophages. T-tests were conducted to compare the differences in pJScav and iBAC(cav)-encoded DNA transfer between Ec(RL123i) and strains harbouring fewer than three lysis genes. Stars indicate significant difference in the DNA transfer between Ec(RL123i) and the other investigated *E*. *coli* strains. The differences were significant (p<0.05) in all cases except when compared to iBAC(cav) transfer in Ec(RL3i) and Ec(RL13i) 2 hours after induction.

To test the suitability of the engineered autolytic *E*. *coli* cells for interchassis DNA transfer we transferred plasmid pJScav and bacterial artificial chromosome iBAC(cav)-borne genetic circuits *cat* and *ven* into *B*. *subtilis*. The genetic circuits *cat* and *ven* encode *Bacillus*-specific chloramphenicol resistance gene and the yellow fluorescent protein-encoding gene *mVenus* flanked by the integration sequences homologous to *amyE* target site in the *B*. *subtilis* chromosome (Fig 4A). To transfer pJScav and iBAC(cav)-borne genetic circuits *cat* and *ven* we used culture mix method [17, 18]. pJScav and iBAC(cav) were first introduced into *E*. *coli* strains Ec(Ri), Ec(RL1i), Ec(RL2i), Ec(RL3i), Ec(RL12i), Ec(RL13i), and Ec(RL123i) by transformation. Temperature shift led to *E*. *coli* cell lysis and release of pJScav and iBAC(cav) from Ec(RL1i), Ec(RL2i), Ec(RL3i), Ec(RL12i), Ec(RL13i), and Ec(RL123i). pJScav and iBAC(cav) were then taken up and incorporated into the *B*. *subtilis* cells cultivated in the same test tube. The resulting recipient *B*. *subtilis* colonies grew on medium containing chloramphenicol and emitted yellow fluorescent light (Fig 4B). This confirmed functionality of the integrated genetic circuits *cat* and *ven* in *B*. *subtilis*. Presence of *amyE* integration target sequences on pJScav and iBAC(cav) led to the integration of genetic circuits *cat* and *ven* into the *amyE* locus of the *B*. *subtilis* chromosome (Fig 4C). Integrations into the *amyE* locus were verified by diagnostic PCR using flanking primers (Fig 4C) and sequencing. The addition of DNAse I into the culture media completely suppressed pJScav and iBAC(cav) transfer from *E*. *coli* to *B*. *subtilis* (Fig 4B), thus confirming that the transfer occurred via stable DNA in the medium. This is in line with the results of the previous study investigating DNA transfer from *E*. *coli* to *B*. *subtilis* by co-culture method [17].

The statistical T-test analysis was performed to compare the differences in the transfer of pJScav and iBAC(cav) between Ec(RL123i) and other investigated strains harbouring fewer lysis genes (Fig 4D). This analysis revealed that there were significant differences in the transfer of pJScav-encoded DNA between Ec(RL123i) and all strains with fewer than three lysis genes both 2 hours [Ec(RL1i) (p<0.001), Ec(RL2i) (p>0.001), Ec(RL3i) (p<0.005), Ec(RL12i) (p<0.05), Ec(RL13i) (p<0.05)] and 6 hours [Ec(RL1i) (p<0.001), Ec(RL2i) (p>0.001), Ec(RL3i) (p<0.005), Ec(RL12i) (p<0.005), Ec(RL13i) (p<0.01)] after induction of the cell lysis. When using iBAC(cav)-encoded DNA, there were significant differences between Ec(RL123i) and all strains with fewer lysis genes 6 hours [Ec(RL1i) (p<0.001), Ec(RL2i) (p>0.001), Ec(RL3i) (p<0.005), Ec(RL12i) (p<0.05) and Ec(RL13i) (p<0.05)] after induction. Two hours after the induction, the differences in iBAC(cav) transfer were significant in most of the strains [Ec(RL1i) (p<0.01), Ec(RL2i) (p>0.05), Ec(RL12i) (p<0.05)] with the exception of Ec(RL3i) and Ec(RL13i).

Notably, in addition to Ec(RL1i), Ec(RL2i), Ec(RL3i), Ec(RL12i), Ec(RL13i), and Ec(RL123i), transfer of pJScav and iBAC(cav) into *B*. *subtilis* occurred even in the absence of any phage gene expression using strain Ec(Ri). This is due to spontaneous lysis of the fraction of *E*. *coli* cell population [43]. DNA released from the spontaneously lysed *E*. *coli* cells can be taken up by *B*. *subtilis* cultivated in the same test tube. The number of transformants using both pJScav and iBAC(cav) was highest with strain Ec(RL123i), thus showing that combining lysis genes from phages MS2, ΦX174, and lambda in a single cell improves transfer of genetic circuits from *E*. *coli* to *B*. *subtilis* (Fig 4D).

## Conclusions

We set out to provide the synthetic biology community with a reliable system for the transfer of genetic circuits from *E*. *coli* to *B*. *subtilis*. This is particularly important for transfer of the high molecular weight DNA molecules, such as BACs, which are prone to mechanical shearing by pipetting [17, 18]. As the presence of an entire phage could be detrimental to the quality of the transferred DNA we aimed to efficiently lyse *E*. *coli* cells using solely the lysis genes from multiple bacteriophages. To this end, we investigated lysis genes from phages MS2, ΦX174, and lambda. Genetic circuits L1, L2, L3, L12, L13 and L123 harbouring different combinations of phage lysis genes were integrated into the *E*. *coli* chromosome using the lambda Red recombineering approach [20]. Our results show that cells harbouring lysis genes from all three bacteriophages lyse with higher efficiency than those with single or two lysis genes. This could be due to the different mechanisms of action of the three phages tested (e.g. phage lambda forms small holes across the cell surface, while MS2 forms a bigger hole at the septal area and φX174 inhibits PG biosynthesis). The engineered autolytic cells allow reliable and efficient transfer of the plasmid and BAC-encoded genetic circuits from *E*. *coli* into *B*. *subtilis*.

To our knowledge, this is the first study investigating synergistic effect of lysis genes from three different bacteriophages on the lysis of *E*. *coli* and DNA transfer from the lysed cells into the *B*. *subtilis* chromosome. Our system does not require the traditional DNA isolation and transformation techniques which are time-consuming. The advantages of our system over other cell lysis methods, such as enzyme degradation and mechanical disruption, particularly on the industrial scale, include lower production and product recovery costs. Furthermore, in our conditionally-inducible cell lysis system there is no need to add an inducer to initiate cell lysis. At the laboratory research scale, transfer of DNA from *E*. *coli* undergoing phage-induced lysis to *B*. *subtilis* by the co-culture method [17, 18] used in our system mitigates against mechanical shearing, which is a serious issue particularly when manipulating the high molecular weight DNA molecules. Previous approaches utilizing an entire phage to lyse the donor *E*. *coli* cells can be detrimental to the quality of the transferred DNA, thus in our system we used solely the phage lysis genes. In the future, this system could be further enhanced by utilization of other lysis genes, such as those of bacteriophages T4, T7, and Mu. Other phage-encoded genes crucial for phage's life cycle could be integrated into the chromosome to improve the kinetics of cell lysis. Alternative promoters, such as *P*_*mgtB*_, *P*_*nisA*_, *P*_*cmp*_ or *P*_*lac*_*-LacI* [16, 44] and the visible light-sensitive lambda cI repressor [45] could be used to fine-tune expression of lysis genes.

## Supporting Information

S1 Fig Plasmids with different combinations of lysis genes. Figure shows confirmation of the plasmids pSB1K3(FRTL1), pSB1K3(FRTL2), pSB1K3(FRTL3), pSB1K3(FRTL12), pSB1K3(FRTL13), and pSB1K3(FRTL123), harbouring different combinations of lysis genes using flanking primers. HyperLadder 1kb (Bioline) has been used as the molecular weight marker.
(TIF)

S1 Table Primers used in this study. (DOC)

## References

[1] Juhas M, Ajioka JW. High molecular weight DNA assembly in vivo for synthetic biology applications. Crit Rev Biotechnol. 2016:1–10. 10.3109/07388551.2016.1141394 . 26863154

[2] Ajikumar PK, Xiao WH, Tyo KE, Wang Y, Simeon F, Leonard E, et al Isoprenoid pathway optimization for Taxol precursor overproduction in Escherichia coli. Science. 2010;330(6000):70–4. 10.1126/science.1191652 20929806 PMC3034138

[3] Harwood CR, Cranenburgh R. Bacillus protein secretion: an unfolding story. Trends Microbiol. 2008;16(2):73–9. 10.1016/j.tim.2007.12.001 . 18182292

[4] Commichau FM, Alzinger A, Sande R, Bretzel W, Meyer FM, Chevreux B, et al Overexpression of a non-native deoxyxylulose-dependent vitamin B6 pathway in Bacillus subtilis for the production of pyridoxine. Metab Eng. 2014;25C:38–49. 10.1016/j.ymben.2014.06.007 . 24972371

[5] Juhas M, Reuß DR, Zhu B, Commichau FM. Bacillus subtilis and Escherichia coli essential genes and minimal cell factories after one decade of genome engineering. Microbiology. 2014;160(Pt 11):2341–51. 10.1099/mic.0.079376-0 . 25092907

[6] Juhas M. On the road to synthetic life: the minimal cell and genome-scale engineering. Crit Rev Biotechnol. 2015:1–8. 10.3109/07388551.2014.989423 . 25578717

[7] Commichau FM, Pietack N, Stülke J. Essential genes in Bacillus subtilis: a re-evaluation after ten years. Mol Biosyst. 2013;9(6):1068–75. 10.1039/c3mb25595f . 23420519

[8] Park SJ, Lee TW, Lim SC, Kim TW, Lee H, Kim MK, et al Biosynthesis of polyhydroxyalkanoates containing 2-hydroxybutyrate from unrelated carbon source by metabolically engineered Escherichia coli. Appl Microbiol Biotechnol. 2012;93(1):273–83. 10.1007/s00253-011-3530-x . 21842437

[9] Yim H, Haselbeck R, Niu W, Pujol-Baxley C, Burgard A, Boldt J, et al Metabolic engineering of Escherichia coli for direct production of 1,4-butanediol. Nat Chem Biol. 2011;7(7):445–52. 10.1038/nchembio.580 . 21602812

[10] Zhou L, Niu DD, Tian KM, Chen XZ, Prior BA, Shen W, et al Genetically switched D-lactate production in Escherichia coli. Metab Eng. 2012;14(5):560–8. 10.1016/j.ymben.2012.05.004 . 22683845

[11] Hao T, Han B, Ma H, Fu J, Wang H, Wang Z, et al In silico metabolic engineering of Bacillus subtilis for improved production of riboflavin, Egl-237, (R,R)-2,3-butanediol and isobutanol. Mol Biosyst. 2013;9(8):2034–44. 10.1039/c3mb25568a . 23666098

[12] Manabe K, Kageyama Y, Morimoto T, Shimizu E, Takahashi H, Kanaya S, et al Improved production of secreted heterologous enzyme in Bacillus subtilis strain MGB874 via modification of glutamate metabolism and growth conditions. Microb Cell Fact. 2013;12:18 10.1186/1475-2859-12-18 23419162 PMC3600796

[13] McKenney PT, Driks A, Eichenberger P. The Bacillus subtilis endospore: assembly and functions of the multilayered coat. Nat Rev Microbiol. 2013;11(1):33–44. 10.1038/nrmicro2921 . 23202530 PMC9910062

[14] Yadav T, Carrasco B, Serrano E, Alonso JC. Roles of Bacillus subtilis DprA and SsbA in RecA-mediated genetic recombination. J Biol Chem. 2014;289(40):27640–52. 10.1074/jbc.M114.577924 25138221 PMC4183802

[15] Shi T, Wang G, Wang Z, Fu J, Chen T, Zhao X. Establishment of a markerless mutation delivery system in Bacillus subtilis stimulated by a double-strand break in the chromosome. PLoS One. 2013;8(11):e81370 10.1371/journal.pone.0081370 24282588 PMC3839881

[16] Gao Y, Feng X, Xian M, Wang Q, Zhao G. Inducible cell lysis systems in microbial production of bio-based chemicals. Appl Microbiol Biotechnol. 2013;97(16):7121–9. 10.1007/s00253-013-5100-x . 23872961

[17] Kaneko S, Itaya M. Designed horizontal transfer of stable giant DNA released from Escherichia coli. J Biochem. 2010;147(6):819–22. 10.1093/jb/mvq012 . 20145021

[18] Itaya M, Kaneko S. Integration of stable extracellular DNA released from Escherichia coli into the Bacillus subtilis genome vector by culture mix method. Nucleic Acids Res. 2010;38(8):2551–7. 10.1093/nar/gkq142 20308163 PMC2860128

[19] Hayashi K, Morooka N, Yamamoto Y, Fujita K, Isono K, Choi S, et al Highly accurate genome sequences of Escherichia coli K-12 strains MG1655 and W3110. Mol Syst Biol. 2006;2:2006.0007 10.1038/msb4100049 16738553 PMC1681481

[20] Juhas M, Evans LD, Frost J, Davenport PW, Yarkoni O, Fraser GM, et al Escherichia coli Flagellar Genes as Target Sites for Integration and Expression of Genetic Circuits. PLoS One. 2014;9(10):e111451 10.1371/journal.pone.0111451 25350000 PMC4211737

[21] Murphy KC, Campellone KG. Lambda Red-mediated recombinogenic engineering of enterohemorrhagic and enteropathogenic E. coli. BMC Mol Biol. 2003;4:11 10.1186/1471-2199-4-11 14672541 PMC317293

[22] Datsenko KA, Wanner BL. One-step inactivation of chromosomal genes in Escherichia coli K-12 using PCR products. Proc Natl Acad Sci U S A. 2000;97(12):6640–5. 10.1073/pnas.120163297 10829079 PMC18686

[23] Gibson D, Young L, Chuang R, Venter J, Hutchison Cr, Smith H. Enzymatic assembly of DNA molecules up to several hundred kilobases. Nat Methods. 2009;6(5):343–5. 10.1038/nmeth.1318 19363495

[24] Merryman C, Gibson DG. Methods and applications for assembling large DNA constructs. Metab Eng. 2012;14(3):196–204. . 22629570 10.1016/j.ymben.2012.02.005

[25] Miller EM, Nickoloff JA. Escherichia coli electrotransformation. Methods Mol Biol. 1995;47:105–13. 10.1385/0-89603-310-4:105 . 7550724

[26] Hanahan D, Jessee J, Bloom FR. Plasmid transformation of Escherichia coli and other bacteria. Methods Enzymol. 1991;204:63–113. . 1943786 10.1016/0076-6879(91)04006-a

[27] Juhas M, Ajioka JW. Identification and validation of novel chromosomal integration and expression loci in Escherichia coli flagellar region 1. PLoS One. 2015;10(3):e0123007 10.1371/journal.pone.0123007 25816013 PMC4376774

[28] Juhas M, Ajioka JW. Flagellar region 3b supports strong expression of integrated DNA and the highest chromosomal integration efficiency of the Escherichia coli flagellar regions. Microb Biotechnol. 2015;8(4):726–38. 10.1111/1751-7915.12296 . 26074421 PMC4476827

[29] Altschul SF, Gish W, Miller W, Myers EW, Lipman DJ. Basic local alignment search tool. J Mol Biol. 1990;215(3):403–10. Epub 1990/10/05. 10.1006/jmbi.1990.9999 S0022283680799990 [pii]. . 2231712

[30] Altschul SF, Madden TL, Schaffer AA, Zhang J, Zhang Z, Miller W, et al Gapped BLAST and PSI-BLAST: a new generation of protein database search programs. Nucleic Acids Res. 1997;25(17):3389–402. Epub 1997/09/01. gka562 [pii]. . 9254694 10.1093/nar/25.17.3389 PMC146917

[31] Young R. Bacteriophage lysis: mechanism and regulation. Microbiol Rev. 1992;56(3):430–81. 1406491 10.1128/mr.56.3.430-481.1992 PMC372879

[32] Berry J, Summer EJ, Struck DK, Young R. The final step in the phage infection cycle: the Rz and Rz1 lysis proteins link the inner and outer membranes. Mol Microbiol. 2008;70(2):341–51. 10.1111/j.1365-2958.2008.06408.x . 18713319 PMC4623567

[33] Catalão MJ, Gil F, Moniz-Pereira J, São-José C, Pimentel M. Diversity in bacterial lysis systems: bacteriophages show the way. FEMS Microbiol Rev. 2013;37(4):554–71. 10.1111/1574-6976.12006 . 23043507

[34] Bienkowska-Szewczyk K, Lipinska B, Taylor A. The R gene product of bacteriophage lambda is the murein transglycosylase. Mol Gen Genet. 1981;184(1):111–4. . 6460914 10.1007/BF00271205

[35] Gründling A, Manson MD, Young R. Holins kill without warning. Proc Natl Acad Sci U S A. 2001;98(16):9348–52. 10.1073/pnas.151247598 11459934 PMC55423

[36] Tanaka S, Clemons WM. Minimal requirements for inhibition of MraY by lysis protein E from bacteriophage ΦX174. Mol Microbiol. 2012;85(5):975–85. 10.1111/j.1365-2958.2012.08153.x 22742425 PMC3429702

[37] Shulman LM, Hindiyeh M, Muhsen K, Cohen D, Mendelson E, Sofer D. Evaluation of four different systems for extraction of RNA from stool suspensions using MS-2 coliphage as an exogenous control for RT-PCR inhibition. PLoS One. 2012;7(7):e39455 10.1371/journal.pone.0039455 22815706 PMC3397973

[38] Nishihara T. Various morphological aspects of Escherichia coli lysis by RNA bacteriophage MS2 observed by transmission and scanning electron microscopes. New Microbiol. 2003;26(2):163–8. . 12737198

[39] Walderich B, Höltje JV. Specific localization of the lysis protein of bacteriophage MS2 in membrane adhesion sites of Escherichia coli. J Bacteriol. 1989;171(6):3331–6. 2656650 10.1128/jb.171.6.3331-3336.1989 PMC210054

[40] Cunningham DS, Koepsel RR, Ataai MM, Domach MM. Factors affecting plasmid production in Escherichia coli from a resource allocation standpoint. Microb Cell Fact. 2009;8:27 10.1186/1475-2859-8-27 19463175 PMC2702362

[41] Nielsen KM, Johnsen PJ, Bensasson D, Daffonchio D. Release and persistence of extracellular DNA in the environment. Environ Biosafety Res. 2007;6(1–2):37–53. 10.1051/ebr:2007031 . 17961479

[42] Gandon S. Why Be Temperate: Lessons from Bacteriophage λ. Trends Microbiol. 2016;24(5):356–65. 10.1016/j.tim.2016.02.008 . 26946976

[43] Corchero JL, Cubarsí R, Vila P, Arís A, Villaverde A. Cell lysis in Escherichia coli cultures stimulates growth and biosynthesis of recombinant proteins in surviving cells. Microbiol Res. 2001;156(1):13–8. 10.1078/0944-5013-00066 . 11372648

[44] Zhang X, Pan Z, Fang Q, Zheng J, Hu M, Jiao X. An auto-inducible Escherichia coli lysis system controlled by magnesium. J Microbiol Methods. 2009;79(2):199–204. 10.1016/j.mimet.2009.09.001 . 19748529

[45] Lee JM, Lee J, Kim T, Lee SK. Switchable gene expression in Escherichia coli using a miniaturized photobioreactor. PLoS One. 2013;8(1):e52382 10.1371/journal.pone.0052382 23349683 PMC3547951

