# Supplementary figures and images for: Combining Genes from Multiple Phages for Improved Cell Lysis and DNA Transfer from Escherichia coli to Bacillus subtilis
Source: PLoS One. 2016 Oct 31;11(10):e0165778. doi: 10.1371/journal.pone.0165778 (PMC5087902; doi:10.1371/journal.pone.0165778)

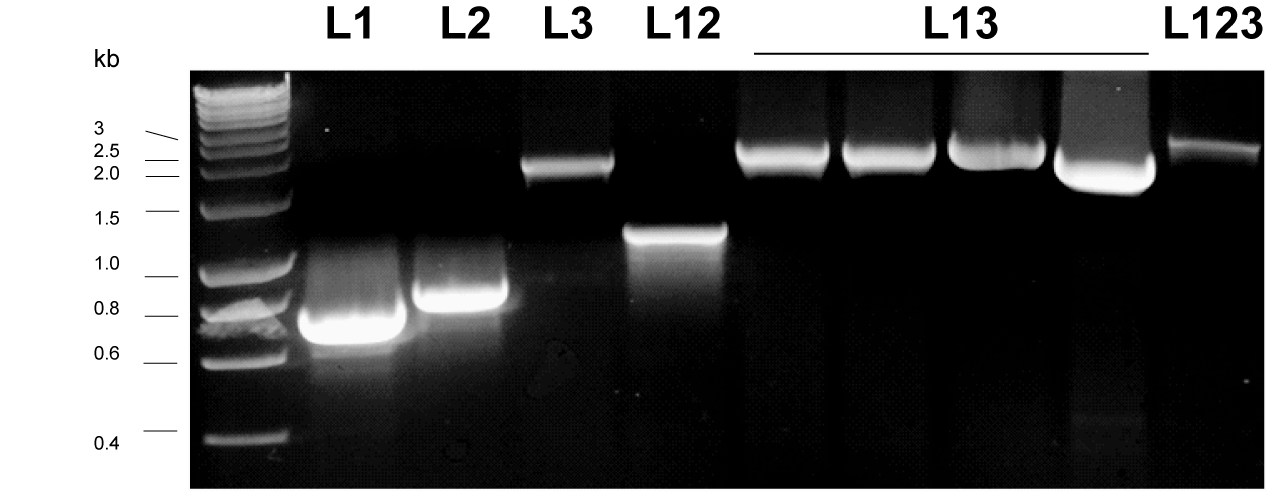

Supplement: S1 Fig — Figure shows confirmation of the plasmids pSB1K3(FRTL1), pSB1K3(FRTL2), pSB1K3(FRTL3), pSB1K3(FRTL12), pSB1K3(FRTL13), and pSB1K3(FRTL123), harbouring different combinations of lysis genes using flanking primers. HyperLadder 1kb (Bioline) has been used as the molecular weight marker. (TIF) [file pone.0165778.s001.tif]
