# Supplementary material for: Combining Genes from Multiple Phages for Improved Cell Lysis and DNA Transfer from Escherichia coli to Bacillus subtilis
Source: PLoS One. 2016 Oct 31;11(10):e0165778. doi: 10.1371/journal.pone.0165778 (PMC5087902; doi:10.1371/journal.pone.0165778)
Supplement: S1 Table — (DOC) [file pone.0165778.s002.doc]

**S1 Table. Primers used in this study.**

**Primer (Sequence 5’→ 3’)**

pL1-1: pSB1K3(FRTL1), pSB1K3(FRTL2), pSB1K3(FRTL3) Gibson Isothermal Assembly primer 1

GTCGACGGATCCCCGGAATTAATTCTCAGTACTATATTCTGGTGCTCACGTGC

pL1-2: pSB1K3(FRTL1), pSB1K3(FRTL2), pSB1K3(FRTL3) Gibson Isothermal Assembly primer 2

GCACGTGAGCACCAGAATATAGTACTGAGAATTAATTCCGGGGATCCGTCGAC

pL1-3: pSB1K3(FRTL1), pSB1K3(FRTL2), pSB1K3(FRTL3) Gibson Isothermal Assembly primer 3

GCTATCGGTAGTCACTTGATGCATCTCCGGCAAAAAAGGGCAAGG

pL1-4: pSB1K3(FRTL1), pSB1K3(FRTL2), pSB1K3(FRTL3) Gibson Isothermal Assembly primer 4

CCTTGCCCTTTTTTGCCGGAGATGCATCAAGTGACTACCGATAGC

pL12-1: pSB1K3(FRTL12) Gibson Isothermal Assembly primer 1

TACAGCAATTGCTTACTTAAATTAAAGAGGAGAAAATGGTACGCTGGACTT

pL12-2: pSB1K3(FRTL12) Gibson Isothermal Assembly primer 2

ACCATTTTCTCCTCTTTAATTTAAGTAAGCAATTGCTGTAAAGTCGTCACTGT

pL12-3: pSB1K3(FRTL12) Gibson Isothermal Assembly primer 3

ACAATTAAAGAGGAGAAAACATGAAGATGCCAGAAAAACATGACCTG

pL12-4: pSB1K3(FRTL12) Gibson Isothermal Assembly primer 4

CTGGCATCTTCATGTTTTCTCCTCTTTAATTCACTCCTTCCGCAC

pL13-1: pSB1K3(FRTL13) Gibson Isothermal Assembly primer 1

CTTAAATTAAAGAGGAGAAAACATGAAGATGCCAGAAAAACATGACCTG

pL13-2: pSB1K3(FRTL13) Gibson Isothermal Assembly primer 2

CATGTTTTCTCCTCTTTAATTTAAGTAAGCAATTGCTGTAAAGTCGTCACTG

pL13-3: pSB1K3(FRTL13) Gibson Isothermal Assembly primer 3

ACAATTAAAGAGGAGAAAACATGAAGATGCCAGAAAAACATGACCTG

pL13-4: pSB1K3(FRTL13) Gibson Isothermal Assembly primer 4

CTGGCATCTTCATGTTTTCTCCTCTTTAATTCACTCCTTCCGCAC

pL123-1: pSB1K3(FRTL123) Gibson Isothermal Assembly primer 1

TACAGCAATTGCTTACTTAAATTAAAGAGGAGAAAATGGTACGCTGGACTT

pL123-2: pSB1K3(FRTL123) Gibson Isothermal Assembly primer 2

ACCATTTTCTCCTCTTTAATTTAAGTAAGCAATTGCTGTAAAGTCGTCACTGT

pL123-3: pSB1K3(FRTL123) Gibson Isothermal Assembly primer 3

ACAATTAAAGAGGAGAAAACATGAAGATGCCAGAAAAACATGACCTG

pL123-4: pSB1K3(FRTL123) Gibson Isothermal Assembly primer 4

CTGGCATCTTCATGTTTTCTCCTCTTTAATTCACTCCTTCCGCAC

L123i-1: Ec(RL123i), Ec(RL1i), Ec(RL2i), and Ec(RL3i) integration primer 1

TATGCTGGCGTCGCTGGACTCTAAACGTATTGATGTGGTGATTAATCAGGTCACCATTTCTGATGAGTATCACGAGGCAGAATTTCAGAT

L123i-2: Ec(RL123i), Ec(RL1i), Ec(RL2i), and Ec(RL3i) integration primer 2

TGTGTTGTTGTGATTTTCTTATTATGCACGCTGAAAACGCGTAAATAAAAAAGGCGCTAGTGAAAGATGCATCAAGTGACTACCGATAGC

fliS-TF: *fliS* integration test primer forward

CCAGGCCTATGCACAAATTGGCGTCG

fliS-TR: *fliS* integration test primer reverse

GAGTAACGACTCTTTCCAGGCATCGGC

fliJ-TF: *fliJ* integration test primer forward

GCTGGCGACCCTGAAAGATCTGG

fliJ-TR: *fliJ* integration test primer reverse

GCCGTTCCTGCAGTGTCTGC

pLYSflank-TF: pSB1K3(FRTL1), pSB1K3(FRTL2), pSB1K3(FRTL3), pSB1K3(FRTL12), pSB1K3(FRTL13), pSB1K3(FRTL123) flanking primer forward

CTGGCTTTCTACGTGTTCCGCTTCC

pLYSflank-TR: pSB1K3(FRTL1), pSB1K3(FRTL2), pSB1K3(FRTL3), pSB1K3(FRTL12), pSB1K3(FRTL13), pSB1K3(FRTL123) flanking primer reverse

TACCGCCTTTGAGTGAGCTGATACC

amyEi-TF: *amyE* integration test primer forward

CCAGTCTTCACATCGGTTTGAAAGG

amyEi-TR: *amyE* integration test primer reverse

ATCATTGATGGTTTCTTTCGGTAAGTCC
